# The Dark Side and the Light Side of Technology-Related Stress and Stress Related to Workplace Innovations: From Artificial Intelligence to Business Transformations

**DOI:** 10.3390/ijerph19031248

**Published:** 2022-01-23

**Authors:** Gabriele Giorgi, Antonio Ariza-Montes, Nicola Mucci, Antonio L. Leal-Rodríguez

**Affiliations:** 1 Department of Human Science, European University of Rome, 00163 Roma, Italy; 2 Management Department, Universidad Loyola Andalucía, 14004 Cordoba, Spain; ariza@uloyola.es; 3 Department of Business Administration, Universidad Autónoma de Chile, Santiago 7500912, Chile; 4 Department of Experimental and Clinical Medicine, University of Florence, Largo Piero Palagi 1, 50139 Florence, Italy; nicola.mucci@unifi.it; 5 Department of Management and Marketing, Universidad de Sevilla, Avda. Ramón y Cajal, 1, 41018 Sevilla, Spain; alleal@uloyola.es

This Special Issue provides new insights into the challenges and opportunities associated with new workplace paradigms and business transformations. Information and communication technologies (ICT) and workplace innovations have permanently modified the structural and social aspects of organizations. In addition, due to COVID-19, the trends of the future of work have undergone strong and irreversible acceleration, with consequences, in terms of new hierarchical forms, the management of tangible and intangible resources, and the use of technologies as an essential element of the workplace. All these features, in turn, pose challenges and risks for the protection of employees’ wellbeing. Indeed, the available data underline the need to rethink management models in order to adapt human resources management (HRM) and occupational health and safety (OSH) practices to the characteristics of the hybrid workplace [1,2,3,4].

The *new way of working* represents an opportunity for organizational research to analyze issues such as social relations, virtual support, new methods of performance evaluation, innovative work behaviors, motivation, job satisfaction, and the evaluation of new psychosocial risks, as in the case of technostress. Thanks to an interdisciplinary approach, the research results could be used to identify feasible interventions to improve the health of individuals and organizations [3,5].

The majority of the articles (13, 72.2% of the total) are empirical studies from different areas of the world (e.g., Spain, Italy, China, Chile, Korea, and the United States), covering several disciplines, such as occupational medicine, organizational psychology, management, social sciences, and technology. Five reviews analyzed the topic of technology in the workplace and the effects of the COVID-19 pandemic, providing a comprehensive overview. Taken together, these studies provide invaluable information on the consequences of workplace innovations and ICT implementation, analyzing possible challenges and opportunities for promoting workers’ wellbeing and performance.

## Overview of Articles in this Research Topic

The manuscripts included in this Special Issue shed light on the worker–workplace innovations relationship, providing useful insights into occupational health promotion. 

The use of technologies in the workplace can lead to more blurred boundaries between work and personal activities. The article by Song et al. [6] investigated cyberloafing behaviors using a sample of 335 Chinese employees. The results show that the observation of cyberloafing is positively associated with perceived norms and cyberloafing behaviors. In addition, perceived norms partially mediate the relationship between observability and employees’ cyberloafing, while the perceived certainty and severity of sanctions moderate the effect of observability on employees’ cyberloafing, as well as the mediating effect of perceived norms.

The use of ICT can have a significant impact on individuals’ wellbeing, both in work and educational contexts. The research by González-López et al. [7] explored the effects of forced digitalization and technostress dimensions on a sample of 337 students, using structural equation modeling based on partial least squares (SEM-PLS). The results show that technostress has negative consequences at the individual, group and professional levels. Furthermore, the research analyzed archetypes of user behaviors.

Technology can also modify communication patterns, thanks to the use of smartphones and online platforms. Moving onto the topic of instant messaging (IM), Jiang et al. [8] used the COR theory to investigate the relationship between IM and psychological withdrawal, and the role of work engagement as a mediator and self-control as a moderator, using the experience sampling method (ESM). The results show that IM played a pivotal role in building social relationships, and that IM demands should not only be considered as a form of distraction.

One of the crucial factors in analyzing the impact of ICT in the workplace is the degree of acceptance, as highlighted by the adoption and use literature. The study by Molino et al. [9] investigated the antecedents (resilience, goal orientation, and opportunities for information and training) and outcomes (work engagement) of technology acceptance in a sample of white- and blue-collar workers, in a total of 598 subjects. The results show that all the indirect effects are significant and highlight the importance of adequate organizational trainings.

Due to the use of ICT, workers can be accessible, even after standard working hours, preventing adequate recovery and psychological detachment. The research by Sandoval-Reyes et al. [10] investigated the relationship between technology use and psychological detachment, considering work overload as a possible mediator. The results support the hypotheses and expand the stressor-detachment model by analyzing the implications of using technologies in the workplace. Clear corporate policies and cultural norms are needed to ensure an adequate balance.

In addition, technology can have different consequences depending on the specific work environment. The research by Estrada-Muñoz et al. [11] investigated the effects of progressive digitalization in the teaching context using a sample of 428 teachers. The authors analyzed technostress, focusing on the following four dimensions: skepticism, fatigue, anxiety, and inefficiency. The results show that 11% of the teachers felt techno-anxious and techno-fatigued, with male teachers showing higher levels of both conditions. Analyzing a population of cruise ship employees, the results of the study by Radic et al. [12] showed that, due to technology and internet communication, workers experience fear of missing important information and a high degree of pressure to always be available. Social pressure and fear of missing out are related to relatedness to friends and family need satisfaction, and internet multitasking, respectively. In addition, relatedness to friends and family need satisfaction has a positive effect on perceived social support, which in turn has a positive effect on life satisfaction. 

Furthermore, structural and technological transformations are altering the nature and organization of work, leading to drastic changes in the economy and market structures. The research by Sanchez-Gomez et al. [13] analyzed the impact of economic stress on innovative work behaviors, considering absenteeism as a possible mediator. On the basis of the conservation of resources (COR) theory and threat-rigidity theory (TRT), the authors tested a mediation model on a sample of 578 employees. The results confirmed the hypotheses and highlighted the importance of economic stress in understanding employees’ performance and withdrawal behaviors.

Another factor that can influence the wellbeing of workers concerns the structure of the work environment. The research conducted by Yu et al. [14] shows that the hotel’s nature-friendly environment reduces burnout, and indirectly influences job satisfaction and job performance. Specially designed green places and existing natural environments decreased negative factors and increased psychological recovery, highlighting the importance of the physical environment not only for a visual effect, but also for psychological comfort. Similarly, the research by Han et al. [15] demonstrated the role of the hotel’s green design in fostering stress resilience, promoting other positive organizational outcomes and creating sustainable hotel buildings.

One of the desirable outcomes for organizations is the satisfaction of their employees. Rodríguez-Cifuentes et al. [16] carried out research to investigate the relationship between job satisfaction and presenteeism, using a sample of 337 subjects. The results highlight that presenteeism is positively associated with job satisfaction, and this relationship is mediated by overcommitment, showing an unexpected path. In addition, work-related bullying moderates these relationships. These findings highlight the need for a work-centered approach that takes into account workers’ perceptions. On the other hand, the study by Fernández-Salinero et al. [17] investigated the impact of group identification on the relationship between job involvement and job satisfaction, and the role of professional skill use as a mediator, using a sample of 420 subjects. The results show that job involvement was strongly related to skill use and group identification, while the interaction between job involvement and group identification was negatively related to skill use. In order to achieve positive organizational results, multiple social identities should be considered.

Work-related stress could also lead to positive outcomes for organizations under certain circumstances. The research by Albort-Morant et al. [18] hypothesized that there is a positive relationship between job stressors and innovation, as hindrance stressors could serve as a motivational source. The results of the study conducted on a sample of 1487 employees show that a lack of job control, job demands, and role ambiguity exert a positive and significant impact on the employees’ levels of innovativeness.

The systematic review by Borle et al. [19] analyzed the association between exposure to techno-stressors and work and health outcomes. The results show that the most frequently analyzed techno-stressors are techno-overload and techno-invasion. In addition, techno-stressors seem to be positively related to work engagement. Moreover, Borle et al. [20] investigated the presence of possible sampling bias in research conducted on work-related technostress, by analyzing the role of socioeconomic position (SEP). The results highlight that in a subsample of 13 studies, 11 studies collected data from workers with higher SEP compared to the general population. Future studies should employ context-sensitive SEP measures to improve the external validity and generalizability.

Bondanini et al. [21] conducted a scientometric analysis to provide a critical review of publications on technostress from 1975 to 2019. Since 2003, there has been exponential growth in technostress production. The results show that only 62 of 143 articles include, in their theoretical references, the perspectives of authors, such as Selye, Lazarus, Karasek, Siegrist and/or Cooper, as the remaining articles adopt a more socio-technical approach.

Two narrative reviews analyzed the topic of COVID-19 in the workplace. The review conducted by Giorgi et al. [22] highlights several negative consequences of the pandemic, such as anxiety, depression, PTSD, suicidal ideas, sleep disorders, and drug and alcohol addiction, that may affect more healthcare workers, especially those on the frontline, migrant workers, and workers in contact with the public. Moreover, job insecurity and uncertainty about the future worsen the psychological condition. The review by Baldassarre et al. [23] investigated the topic of stigma and discrimination associated with COVID-19. The results highlight that stigmatization associated with infectious pathologies can significantly affect patients’ and healthcare workers’ psychological wellbeing. A multidisciplinary approach is needed to avoid the negative consequences of discrimination and promote occupational health.

## Conclusions

The results of the manuscript contained in this Special Issue provide an interdisciplinary overview of the issues that form the fabric of the future of work, using data based on a cumulative sample of over 6200 workers. Companies need to implement evidence-based strategies and policies to ensure healthy workplaces. Several factors can shape employees’ experiences in the post-pandemic world of work, such as ICT, economic conditions, and hybrid models. In this regard, we believe that a virtuous circle between scientific literature and organizational practices is one of the key elements for obtaining sustainable organizations.

In conclusion, we would like to stress the mutual relationship between the human (psychophysical health) and the economic capital of organizations. Indeed, ensuring decent working conditions and healthy work environments leads to positive consequences at the individual and business levels. This same principle is stated in the *motto* of the Business@Health Laboratory of the European University of Rome, “*business doesn’t exist without workers’ health and workers’ health is business.*”

## Data Availability

Not applicable.
